# Antibacterial, Structural and Optical Characterization of Mechano-Chemically Prepared ZnO Nanoparticles

**DOI:** 10.1371/journal.pone.0154704

**Published:** 2016-05-16

**Authors:** Umair Manzoor, Sumera Siddique, Rafay Ahmed, Zobia Noreen, Habib Bokhari, Iftikhar Ahmad

**Affiliations:** 1 Alamoudi Water Research Chair, King Saud University, Riyadh, Kingdom of Saudi Arabia; 2 Center for Micro and Nano Devices (CMND), Department of Physics, COMSATS Institute of Information Technology, Islamabad, Pakistan; 3 School of Systems and Technology, University of Management and Technology, Lahore, Pakistan; 4 Department of Bio Sciences, COMSATS Institute of Information Technology, Islamabad, Pakistan; 5 Center of Excellence for Research in Engineering Materials (CEREM), Advanced Manufacturing Institute (AMI), King Saud University, Riyadh, Saudi Arabia; Institute for Materials Science, GERMANY

## Abstract

Structural investigations, optical properties and antibacterial performance of the pure Zinc Oxide (ZnO) nanoparticles (NPs) synthesized by mechano-chemical method are presented. The morphology, dimensions and crystallinity of the ZnO NPs were controlled by tweaking the mechanical agitation of the mixture and subsequent thermal treatment. ZnO nanoparticles in small (< 20 nm) dimensions with spherical morphology and narrow size distribution were successfully obtained after treating the mechano-chemically prepared samples at 250°C. However, higher temperature treatments produced larger particles. TEM, XRD and UV-Vis spectroscopy results suggested crystalline and phase pure ZnO. The NPs demonstrated promising antibacterial activity against Gram negative foodborne and waterborne bacterial pathogens i.e. Enteropathogenic *E*. *coli* (EPEC), *Campylobacter jejuni* and *Vibrio cholerae* as well as Gram positive methicillin resistant *Staphylococcus aureus* (MRSA), thus potential for medical applications. Scanning electron microscopy and survival assay indicated that most probably ZnO nanoparticles cause changes in cellular morphology which eventually causes bacterial cell death.

## Introduction

Contemporary research on Zinc oxide (ZnO) nanoparticles (NPs) has shown their potentials for optical devices (UV absorption), chemical engineering (NO_X_ decomposition, gas sensors, catalyst, pigments, deodorization etc.) and medicine technology (antibacterial treatment) [1–8]. In this context, the preparation of ZnO nanomaterials in small and even dimensions is imperative and several techniques based on chemical or physical methods have been practiced including mechanical agitation [9–13], thermal hydrolysis technique [14], hydrothermal processing [15], sol–gel method [16] spray pyrolysis [17], thermochemical/flame decomposition of metal-organic precursors [18] and thermal evaporation [19]. Amongst these mechanical milling has proved to be an effective and simple technique to produce nanocrystalline powders. Mechano-chemical or solid-state reactions are particularly suitable for the large-scale production of NPs because of their simplicity and low cost [20]. Since such reactions do not involve organic solvents therefore, for controlling the nucleation and growth of NPs, they are attractive, from an environmental point of view [21].

Advent of infectious diseases and emergence of antibiotic resistant bacterial strains are the foremost challenge for public health. Nevertheless, conventional antibiotics are still controlling the bacterial infections resulting from both community and hospital environments however; most of them are ineffective against new bacterial species and gaining resistance against existing antibiotic. Therefore, new technologies are imperative to combat these contemporary threats to humankind and environment [22,23]. Recently developed nanomaterial system has been evaluated for utilizing their best potentials for nanobiotechnology and it is thought that the promising traits of these tiny materials can be helpful in upgrading the contemporary antibacterial system. In this context, metal oxide NPs have received great attention due to their unique physical, chemical, and biological properties and interestingly these properties may be altered by tuning their structure and morphology at atomic level [24–25]. Nanomaterials based antibacterial agents are anticipated revolutionary for food technology and human pathogenic bacteria such as Escherichia coli and Staphylococcus aureus [26–27] Thus, bactericidal properties investigation of the nanomaterial especially ZnO NPs is amongst the current hot research programs [28].

This report presents the preparation of ZnO NPs by mechano-chemical technique and contribution of thermal treatments on their dimensions and morphology. The main aims are to find the role of small dimensions with narrow size distribution on the structural features and optical properties. The suitability of mechano-chemically prepared/thermally treated ZnO NPs, as potential antibacterial material, for medical applications was also evaluated.

## Experimental Procedure

Anhydrous ZnCl_2_ and anhydrous Na_2_CO_3_ were the base starting materials and NaCl was used as a diluent for ZnO NPs synthesis. The stoichiometric mixture of the starting powders was milled in a planetary milling machine (pulverisette 5, FRITSCH), according to the reaction given in Eq (1);

$$\text{Zn}{\text{Cl}}_{2}+{\text{Na}}_{2}{\text{CO}}_{3}+8.6\text{NaCl}\to {\text{ZnCO}}_{3}$$ (1)

Mechano-chemical milling was carried out in 500 ml steel jar using stainless steel (ϕ 10 mm) grinding media at 250 rpm milling speed for the duration of 25 h and the grinding media to powder ratio was set at 16:3. After grinding process, the powder was divided into 4 parts and subsequently treated at different temperatures for 30 minutes in air (Table 1). A mixture of ZnO NPs and NaCl was produced upon heat treatment of the ZnCO_3_ and the thermal decomposition reaction is shown in Eq (2).

$$\text{Zn}{\text{CO}}_{3}\to \text{ZnO}+{\text{CO}}_{2}$$ (2)

**Table 1:** Summary of crystallite size of ZnO NPs estimated using Scherrer’s equation.

Sample ID	Calcination temperature (°C)	Mean crystallite size (XRD) (nm)	Mean particle size (TEM) (nm)	Band gap (eV)
S1	250	19.4	< 20 ± 5	3.29
S2	300	27.6	< 30 ± 6	3.28
S3	350	28.9	< 50 ± 9	3.28
S4	400	30.7	> 60 ± 8	3.27

A tube furnace (Termolab, TH1600) was used for thermal treatment. After thermal treatments, all samples were thoroughly washed several times and carefully filtered with distilled water in order to separate ZnO NPs from NaCl. At the end, obtained ZnO NPs were rinsed with ethanol to remove any unwanted contaminations and also to avoid water related agglomeration. High resolution transmission electron microscope (TEM, Hitachi HNAR9000) was opted to study the morphology and dimensions of the prepared ZnO NPs. The ZnO NPs were structurally appraised by X-ray diffraction (XRD, X'Pert, PANalytical, Cu-Kα radiations) and the bandgap energy was estimated employing UV-Vis reflectance spectroscopy (Lemda-950 Perkin-Elmer).

The synthesized ZnO nanoparticles were screened for their antibacterial activity using agar-well diffusion method [29] against clinical waterborne and foodborne gram negative bacteria (Vibrio cholerae, Enteropathogenic Escherichia coli, Campylobacter jejuni) and gram positive bacteria (methicillin resistant Staphylococcus aureus (MRSA)). All the isolates were obtained from Microbiology and Public health lab, COMSATS Institute of Information Technology Islamabad, Pakistan. Bacteria were routinely cultivated overnight in Luria Bertani Broth medium at 37°C (with the exception of Campylobacter jejuni which is grown for 48hrs at 42°C under microaerophilic conditions). Different concentrations of the test substances (100 μL) dissolved in DMSO were added in well i.e., 0.78 mM, 1.56 mM, 3.125mM, 6.25mM, 12.5 mM, 25 mM, 50 mM. Ampicillin (12.8 mg/mL) and DMSO were used as positive and negative control respectively. Micro-titer assay method was employed to find the minimum inhibitory concentration (MIC) values. 2,3,5-triphenyltetrazolium chloride (TTC) was used as indicator solution [30]. 20 ml of this solutions was then added in the related well (96-well plates). After that, 180 μl of bacterial suspension (107 CFU/ml) was added with final concentration of 0.0786 mM, 0.156 mM, 0.3125 mM, 0.625 mM, 1.25 mM, 2.5 mM, 5.0 mM incubated at 37°C for 24 hrs (48hrs at 42°C for C. jejuni). After incubation, 20μl of TTC indicator solution (5mg/ml) was added followed by reincubation for 15–20 minutes. No change in color was a clear indication of dead or inactive bacteria and a color change towards pink was a clear sign of the presence of viable bacteria. The average of three readings was taken as MIC of nanoparticles against specific bacteria.

To quantitatively analyze the effect of ZnO on bacterial cell overnight culture of the test bacteria were used to inoculate LB media (~10^7^ CFU) with and without addition of synthesized nanoparticle (concentration twice less than MIC). The culture flasks were placed in shaker incubator (100rpm) for 4hours at 37°C (42°C *C*. *jejuni*). After the specified time samples were drawn and serially diluted. The dilutions were spotted on Muller Hinton Agar plate and after 24 hrs of incubation colonies were counted in term of CFU. Un-inoculated media with nanoparticles was used as negative control. The survival percentage was calculated by using the following formula (Eq 3) [31].

$$\%\;\text{Survival}=\frac{\text{Number of colonies in treatedsamples}}{\text{Number of colonies in control samples}}\;\text{x}100$$ (3)

To investigate the morphology changes induced by ZnO one Gram positive and on Gram negative bacteria i.e., *E*. *coli* and *S*. *aureus* were selected for scanning electron microscopy (SEM). Mid-log-phase bacterial cultures were treated with ZnO nanoparticles (concentration 2× less than MIC) for 4h under. The cell suspensions were fixed with a primary fixative solution containing 2.5% glutaraldehyde in PBS buffer solution (pH 7.2) overnight. Subsequently, the fixative solution was exchanged for PBS buffer, followed by dehydration with a series of ethanol solutions (50%, 70%, 90% and 100%), with three ethanol changes at each concentration. The suspension was place on cover slips and which were then gold coated before mounted on stubs with carbon adhesive tabs for SEM.

## Results and Discussion

**Fig 1:**
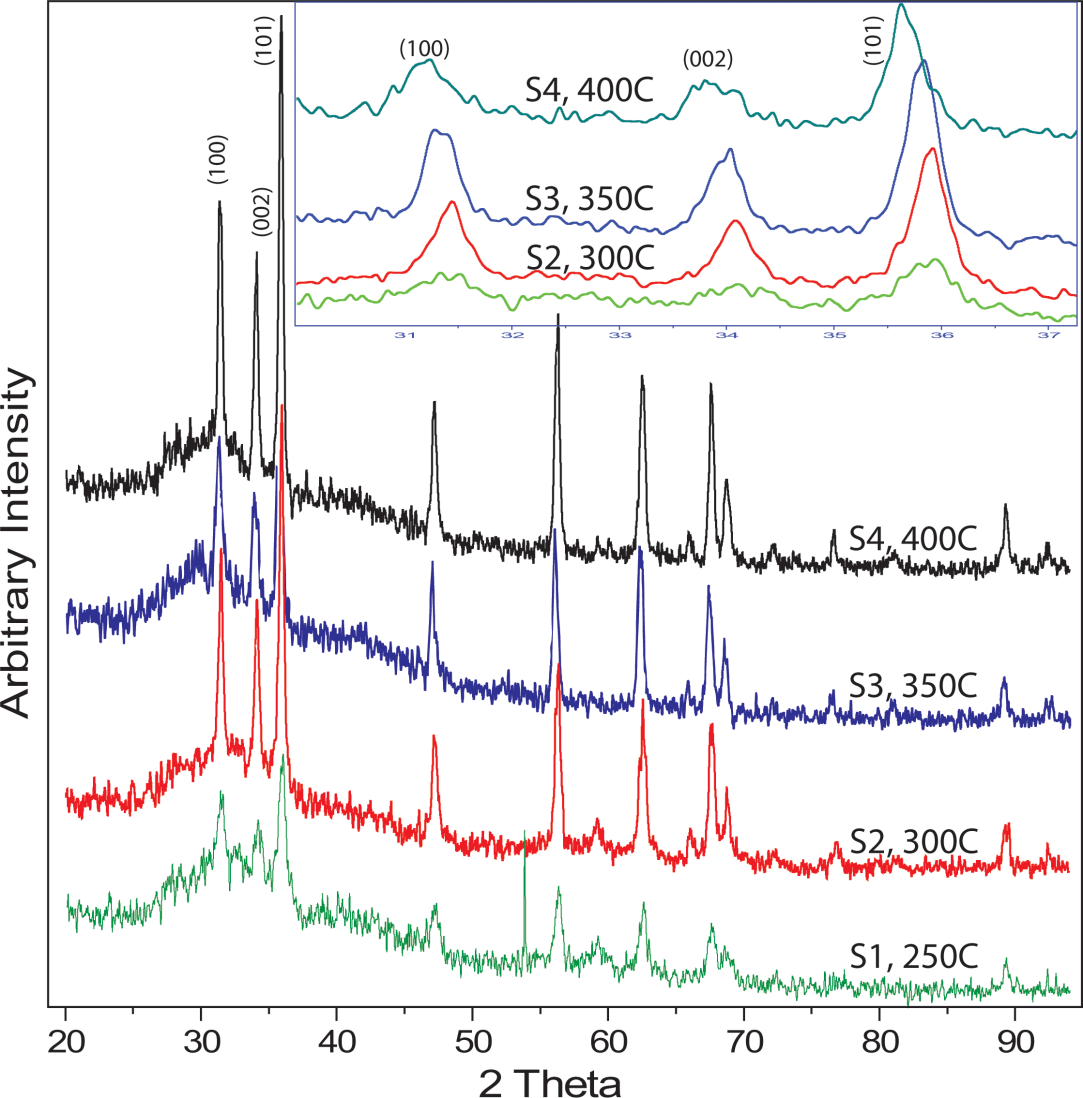
XRD peaks of ZnO NPs calcined at different temperatures suggests an improvement in the FWHM with the increase in the calcination temperature. The inset is the same XRD data showing only major peaks between 2 theta 30~37. The results suggested a systematic shift of (101) peak towards lower angles.

Fig 1 manifests the XRD patterns of thermally treated ZnO NPs. All major peaks are labelled and correspond to hexagonal Wurtzite structure. The highest intensity peak was (101) according to the (JCPDS Card # 65–3411). Fig 1 also confirms the complete removal of the NaCl by-product phase, as only ZnO peaks exist. Furthermore, sharp diffraction peaks were observed for samples calcined at higher temperatures. With the increase in calcination temperature, a systematic shift towards a lower 2 theta was also observed and this shift may be associated with the alteration in the crystal interlammer ‘*d*’ spacing. Thermal treatment of ZnO NPs significantly affected the crystal structure dimensions, which was estimated by employing Scherrer’s equation [28]. It is evident that the crystallite size increases rapidly from about 19.4 nm to 27.6 nm when calcined at 300°C (Table 1), but increases slowly above 300°C and reached 30.7 nm at 400°C.

**Fig 2:**
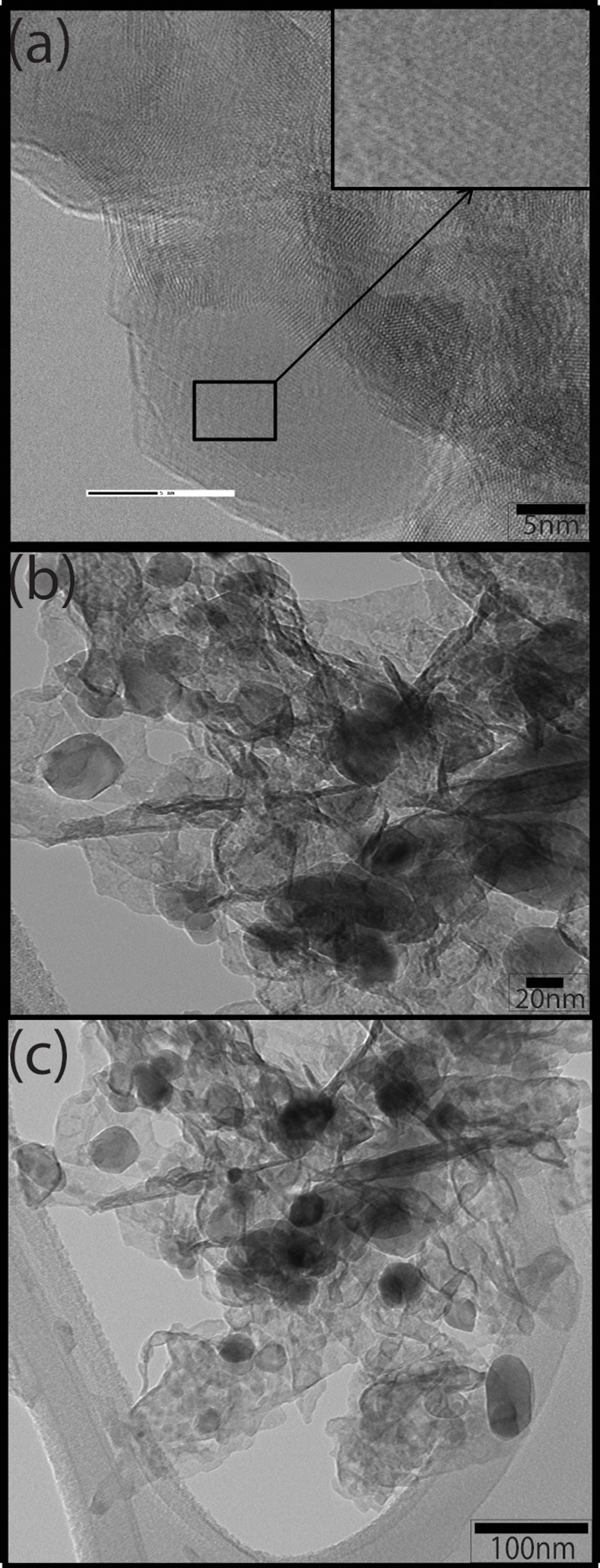
(a) TEM micrographs of ZnO NPs after thermally treatment at (a) 250°C and inset is HR-TEM image showing atomic fringes (b) 300°C (c) 350°C and (d) 400°C.

Fig 2 shows the TEM images of ZnO NPs thermally treated at dissimilar temperatures. It is clear from Fig 2A that the synthesized ZnO NPs are single crystal particles (Inset Fig 2A), with moderate agglomeration and reasonably narrow particle size distribution. It was also noted that the size of ZnO NPs increases with rising calcination temperatures and at 300°C and above exhibits clear crystal faceting suggesting better crystalinity. Also, the particle size and the size distribution are much lower than the already reported by mechanochemical reaction [13] Furthermore, particle size measurements from TEM images are higher than obtained from XRD (Scherrer’s formula). The difference in the particle size obtained from both techniques may be due to the following reasons (i) Scherrer’s formula is originally developed for cubic structures and the values usually don’t match with the actual sizes for other structures, (ii) Scherrer’s formula measures crystallite size while TEM measures the particle size and (iii) usually X-rays are not perfectly parallel when shine on the sample and the reflections are also have the same effect, resulting in peak broadening which ultimately results in smaller crystallite size than the actual.

**Fig 3:**
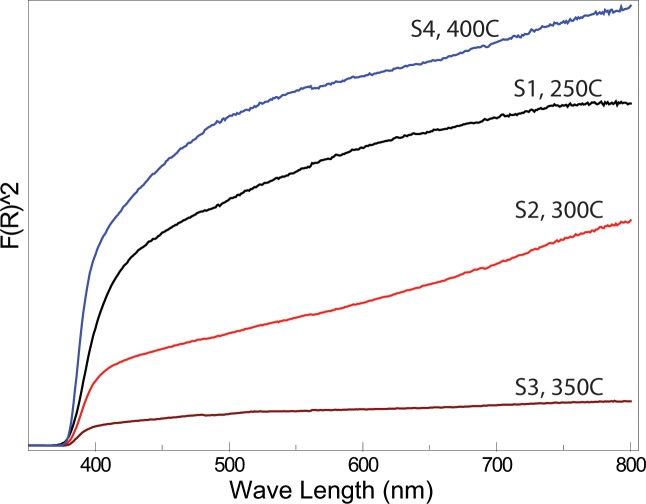
(a) Kubelka-Munk function “F(R)^2^” plotted against wavelength (nm). Band gaps of ZnO NPs were also calculated.

Optical characterization can give an insight into the quantum confinement effects and measure the bandgap [29]. The UV-vis spectroscopy results of all samples are shown in Fig 3. The Kubelka-Munk function F(R) was calculated by following relation.

$$\text{F}(\text{R})=\frac{{\left(1-\text{R}\right)}^{2}}{2R}$$ (4)

Table 1 also shows the bandgap values of all samples and a slight change with respect to particle size can be seen. The results suggested that the energy slightly decreases as the particle size increases (Table 1). Also, UV-Vis spectroscopy shows a sharper slope, with increase in particle size, suggesting lower crystal defects. Also, the smaller NPs have larger surface to volume ratio and therefore the carriers are confined in a very small space. The movements of electrons and holes are confined in a potential well (quantum confinement) [32–33]. This can lead to stronger coupling interaction and probability of binding can increase.

TEM, XRD and UV-Vis spectroscopy results are in good agreement with each other. With the increase in calcination temperatures, TEM images shows faceted morphology, XRD peaks suggested a systematic peak shift towards lower angle which are closer to bulk ZnO values, and UV-Vis spectroscopy show a sharper slope. All these results suggest decrease in crystal defects with increase in the particle size.

**Fig 4:**
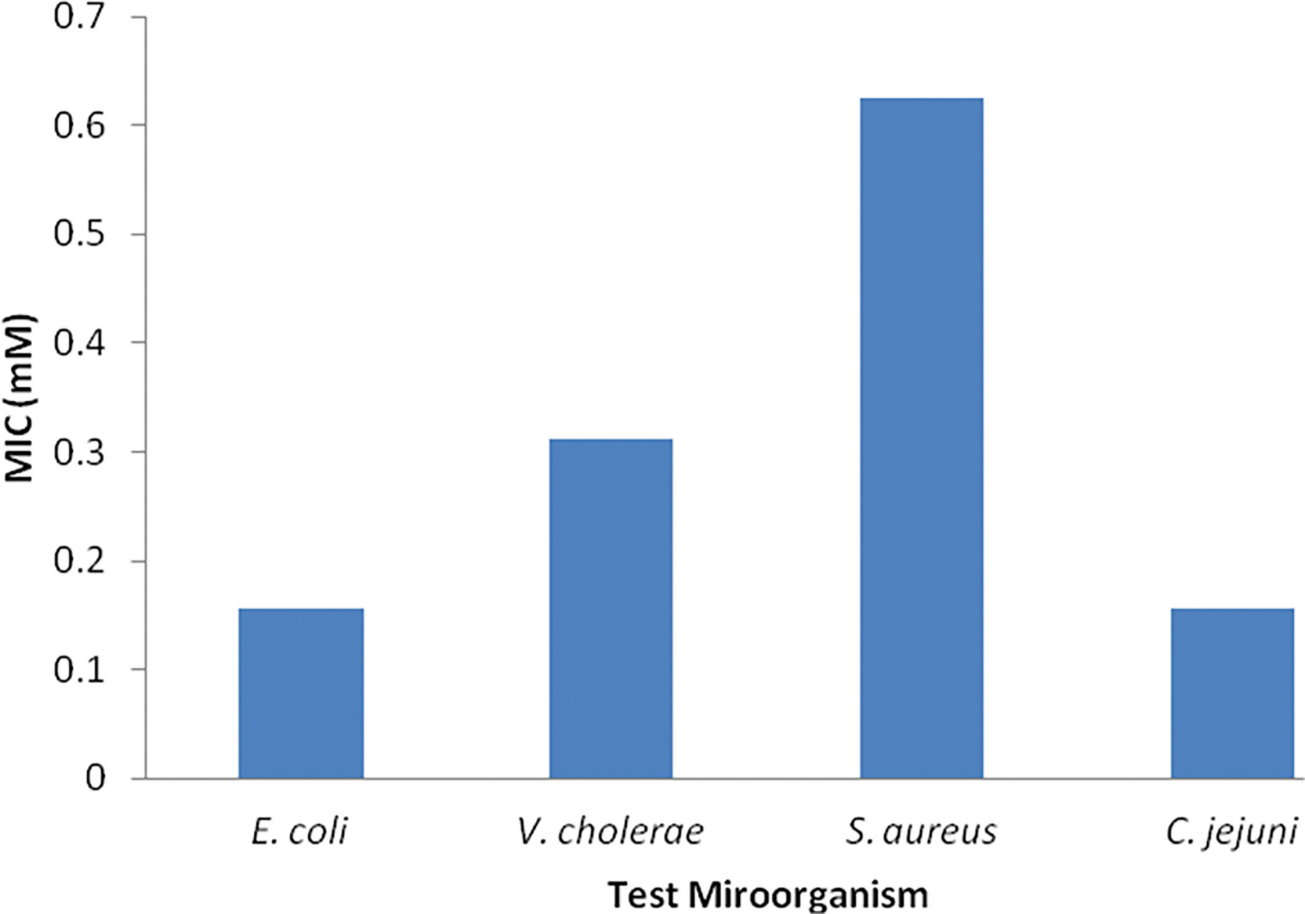
Antibacterial potential (minimum inhibitory concentration) of synthesized ZNO nanoparticles

**Fig 5:**
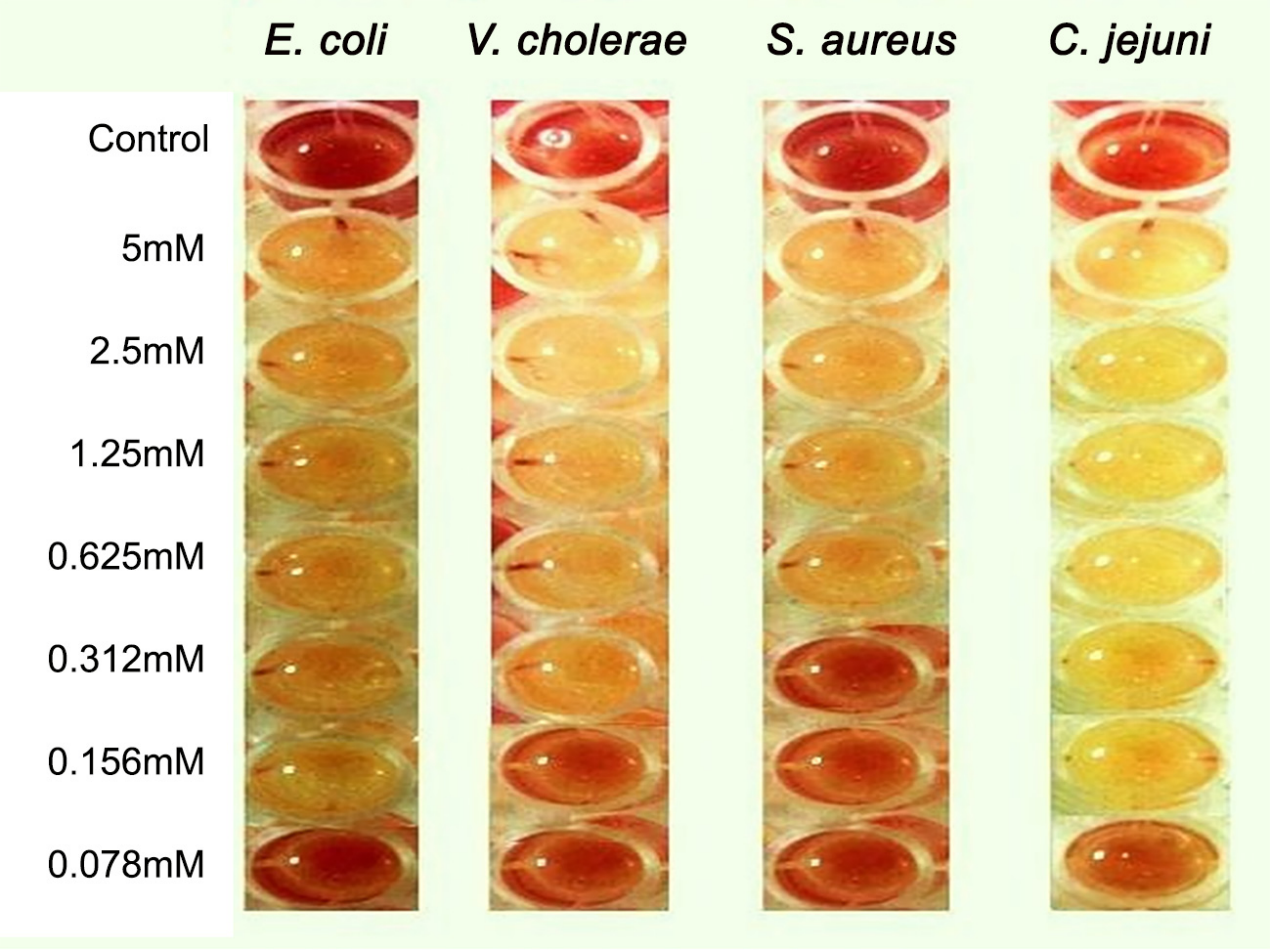
MIC of ZnO nanoparticles using tetrazolium chloride based micro-dilution method.

The as-synthesized sample S3 was tested for antimicrobial activity against three Gram negative bacteria (Enteropathogenic *Escherichia coli*, *Campylobacter jejuni* and *Vibrio cholera)*, and one gram positive bacteria (methicillin resistant *Staphylococcus aureus*). The results showed that NPs were effective against all tested microorganism (Figs 4 and 5) hence showing broad spectrum activity. However, the nanoparticles were most effective against *Campylobacter jejuni* as shown by low MIC (0.156mM) and Enteropathogenic *Escherichia coli* (0.156mM), *Vibrio cholerae* (MIC = 0.312mM) and *Staphylococcus aureus* (0.625mM).

**Fig 6:**
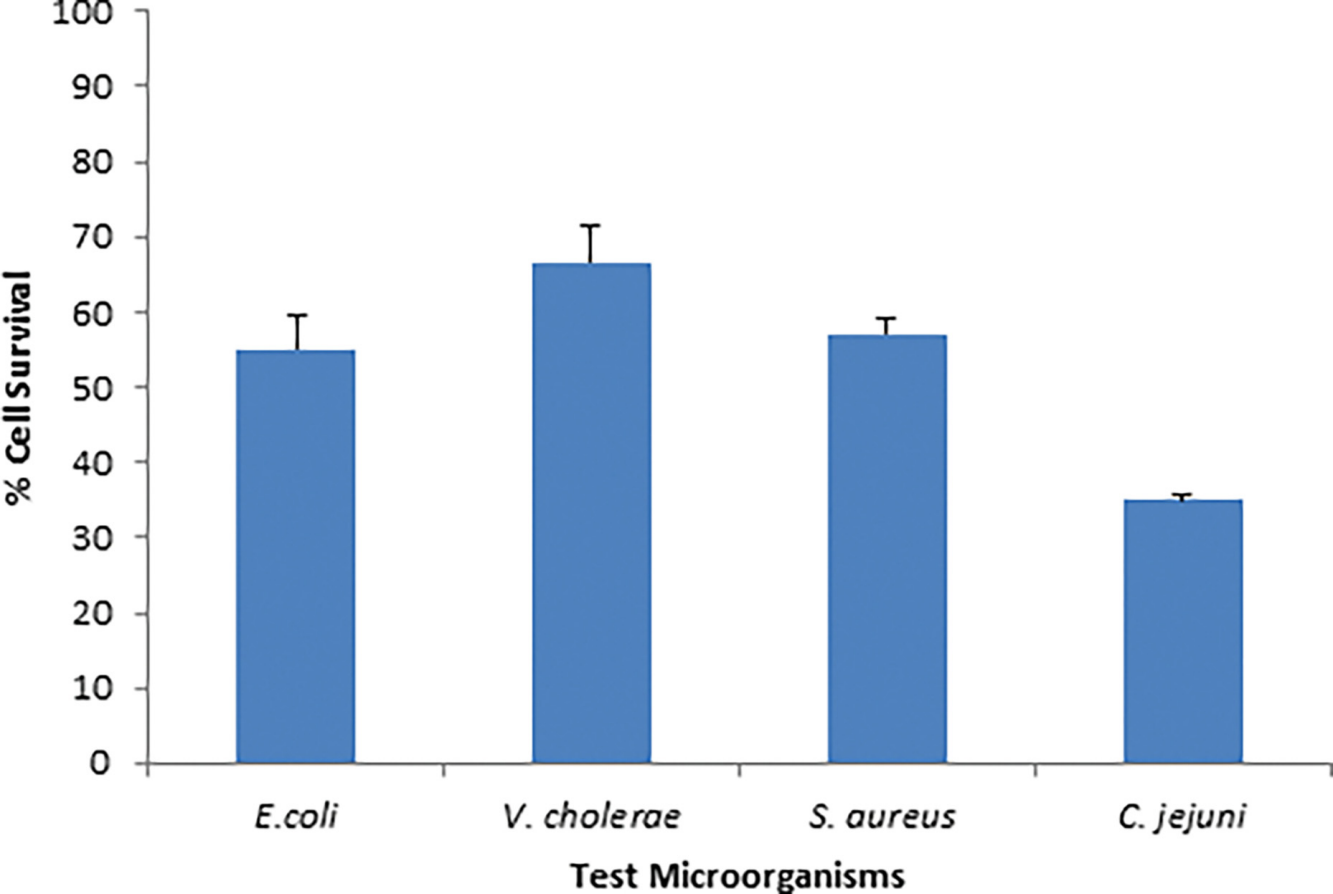
Percentage of Cell survival in response to exposure of ZnO nanoparticles.

In response to exposure of ZnO nanoparticles for 4hrs significant reduction percentage of cell survival was observed against all bacteria (p≤0.005). The lethal effect of ZnO was most effective against *C*. *jejuni* and least effective *V*. *cholerae* as indicated by decrease in viable cell to 35% and 67% respectively on among all tested bacteria (Fig 6).

**Fig 7:**
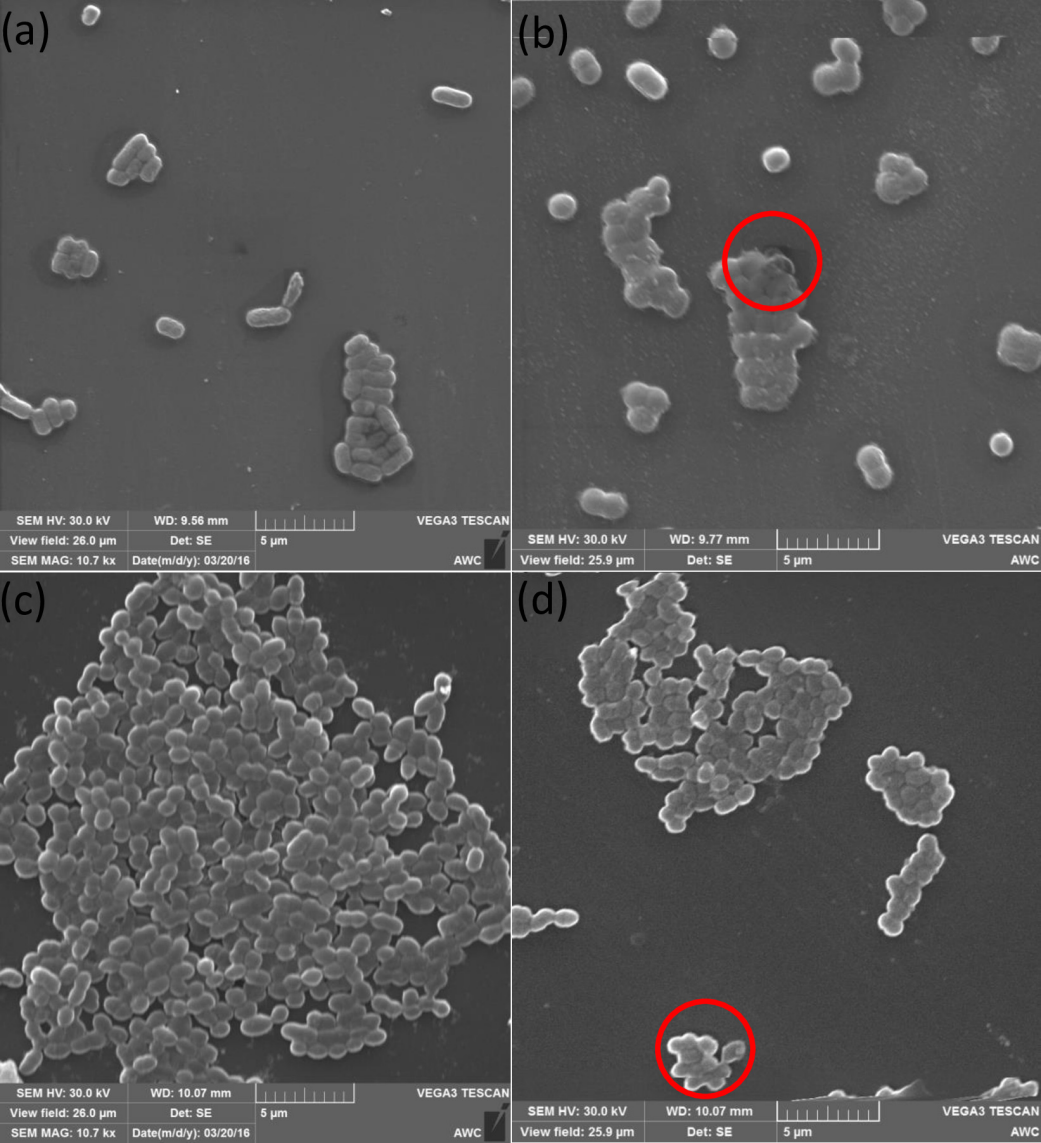
SEM images of E. coli and S.aureus. (a) E. coli cells untreated (b) E. coli cells treated with ZnO nanoparticles for 4 h. (c) S.aureus cells untreated (d) S.aureus cells treated with ZnO nanoparticles for 4 h.

The Effects of ZnO nanoparticles on cellular morphology of *E*. *coli* and *S*. *aureus* were examined by SEM. The SEM micrographs of both control and treated *E*. *coli* and *S*. *aureus* cells are shown in Fig 7. As shown in Fig 7A and 7C untreated *E*. *coli* and *S*. *aureus* showed intact cells and normal morphology i.e., rod-shaped and round-shaped, respectively. After exposure to ZnO nanoparticles for 4 h, the irregular cell surface and leakage of cell was observed in case of *E*. *coli* (Fig 7B) was and whereas in case of *S*. *aureus* leakage of cellular components in some cells was observed as highlighted in Fig 7D. Together these survival assay and SEM results suggest that the ZnO NPs not only induced changes in cellular morphology but also cause a lethal effect against the tested bacteria.

There are many proposed mechanisms for ZnO NPs action against bacteria. For example, nanoparticles attachment to bacterial cell wall via hydrophobic, electrostatic, and receptor-ligand interactions and van der waals forces which leads to cellular damage and eventual death. Also, ZnO NPs generate reactive oxygen species such as H_2_O_2_. There is an interaction between cell membrane and H_2_O_2_. It has an influence on cell physiological activities by interacting with the cell membrane bilayer and influencing integrity, membrane fluidity, and lateral organization [30, 34–36]. These potential effects can be some of the reasons for NPs cytotoxicity.

There are many proposed interaction mechanisms of ZnO NPs to bacterial cells, as mentioned earlier. Important bacterial biomolecules can also adsorb on ZnO NPs. Also, protein structural changes and phospholipid molecular damage are more likely reasons for bacterial toxicity. Toxicity of NPs is probably due to the dissolved metal ions and from NPs tendency to interact with the cell walls. Also, some very basic questions i.e. (i) the toxicities are due to nanosize or just because of composition? (ii) main mechanism of cell damage in NPs-cell surface interactions and (iii) do the morphology and size of particles also play a role in bacterial adhesion etc, these paradigms still need a deep understanding and in-depth knowledge.

## Conclusions

Pure ZnO NPs were prepared successfully by mechano-chemical route. Size of the NPs was controlled by carefully changing the heat treatment temperatures. TEM, XRD and UV-Vis spectroscopy results are in good agreement with each other. TEM images show faceted morphology, XRD peaks suggested a systematic peak shift towards lower angle which are closer to bulk ZnO values, and UV-Vis spectroscopy show a sharper slope. All these results suggested decrease in crystal defects with increase in the particle size. Sample S3 showed broad spectrum activity against different gram negative and gram positive bacteria. However, the nanoparticles were most effective against *Campylobacter jejuni*. Both cell viability assay and SEM analysis showed that the exposure to ZnO leads to change in cellular morphology and eventual death of the bacteria. These finding suggested that ZnO NPs prepared by mecho-chemical route can be effective and have potential for medical applications.
